# Thoracoscopic enucleation of a giant horseshoe-shaped esophageal leiomyoma

**DOI:** 10.1016/j.ijscr.2025.111257

**Published:** 2025-04-04

**Authors:** Motoki Murakami, Yoshinori Ishida, Yasunori Kurahashi, Hisashi Shinohara

**Affiliations:** Department of Gastroenterological Surgery, Hyogo Medical University, 1-1 Mukogawa-cho, Nishinomiya, Hyogo 663-8501, Japan

**Keywords:** Thoracoscopic enucleation, Giant esophageal leiomyoma, Horseshoe-shaped leiomyoma, Esophageal tumor, Video case report

## Abstract

**Introduction:**

Leiomyomas are the most common esophageal benign tumors. Interventions are undertaken if these are large or actively growing or the patients are symptomatic. Though more invasive than enucleation, esophagectomy has been performed in several giant esophageal leiomyoma cases because enucleation requires advanced surgical skills.

**Presentation of case:**

A 42-year-old woman presented with an abnormality on chest radiography. Although she was asymptomatic, enhanced computed tomography revealed a 10-cm solid homogeneous tumor in the lower thoracic esophagus. Esophagogastroscopy visualized the submucosal tumor without mucosal invasion. Endoscopic ultrasonography revealed that the tumor was almost entirely circumferential in the submucosal layer. Biopsy examination revealed a leiomyoma, and thoracoscopic enucleation was attempted.

Right-sided thoracoscopy was performed in the prone position. The giant tumor in the lower thoracic esophagus, which did not invade the neighboring organs, was identified easily and exposed by separating the esophageal muscle layer overlying it. The tumor was difficult to detach from the left side of the esophagus; therefore, Endoloop™ (Johnson & Johnson, NJ, USA) was used for traction without risking tumor damage. After successful tumor enucleation, the muscle layer was closed without stenosis by suturing.

**Discussion:**

As tumor cell dissemination due to messy tumor handling must be avoided, the Endoloop was very useful for maneuvering the tumor on the narrow surgical field. A key element of esophageal tumor enucleation was proper preservation of the mucosa and muscular layer.

**Conclusion:**

Minimally invasive thoracoscopic enucleation may be a treatment option for even large esophageal leiomyomas.

## Highlights

• Leiomyomas are the most common esophageal benign tumors.
• Complete enucleation of a horseshoe-shaped esophageal leiomyoma was performed.
• Minimally invasive enucleation may be a treatment option for esophageal leiomyomas.

## 1 Introduction

Leiomyomas, the most common esophageal benign tumors [1], arise from smooth muscle cells in the esophageal wall. Interventions are undertaken if these are large or actively growing or the patient is symptomatic. Though more invasive than enucleation, esophagectomy has been performed in several giant esophageal leiomyoma cases because enucleation requires advanced surgical skills [[2], [3], [4]], especially for tumors of >8 cm [1,5].

We present a successful case of complete thoracoscopic enucleation of a large horseshoe-shaped esophageal leiomyoma, accompanied by a short video. The work has been reported in line with the SCARE criteria [6].

## 2 Presentation of case

A 42-year-old woman presented to our hospital owing to an abnormal finding during chest X-ray examination. Although she was asymptomatic, computed tomography revealed a 10-cm horseshoe-shaped tumor in the lower thoracic esophagus. Endoscopic ultrasonography demonstrated that the tumor was submucosal and biopsy examination helped establish a pathological diagnosis of a leiomyoma. We decided to perform surgery because it could become more difficult if the tumor were to grow further. Right-sided thoracoscopic enucleation was performed.

Under general anesthesia, a right-sided thoracoscopy was performed with the patient in the prone position. A 12-mm camera port was placed in the 9th intercostal space at the line with the inferior tip of the right scapula and another 12-mm working port was placed in the 7th intercostal space at the posterior axillary line. Two 5-mm ports were located in the 5th intercostal space at the posterior axillary line and the 7th intercostal space at the mid-axillary line. During the operation, an additional 5-mm port was added in the 7th intercostal space near the line of the inferior tip of the right scapula.

The tumor was located in the lower esophagus. First, the right pulmonary ligament was resected. Second, the right mediastinal pleura and muscular layer of the esophagus were separated to expose the tumor. We proceeded to detach the tumor from the head side, keeping the outer membrane of the tumor intact. Then, the proximal side of the tumor was encircled and a gauze was used for traction. The tumor was detached from the muscular and mucosal layers of the esophagus on the dorsal and caudal sides. Thereafter, Endoloop™ was applied to the tumor for traction. This allowed us to avoid direct grasping and facilitated better maneuverability of the tumor. The left side of the tumor was also detached continuously, and the tumor was successfully excised without incising the mucosal layer of the esophagus. Then, the muscular layer was closed using absorbable 3–0 monofilament sutures. Intraoperative endoscopy confirmed no strictures or fragility of the esophagus. Finally, the pleura was closed following an endoscopic leak test. The tumor was placed in a plastic bag and removed through a 5 cm incision by rotating it along its curvature.

The postoperative course was uneventful, and the patient was discharged on postoperative day 14. Fortunately, she experienced no respiratory or circulatory complications after the removal of the massive tumor. The final histopathological diagnosis was leiomyoma of the esophagus. In the postoperative follow-up, special attention was paid to esophageal stricture due to circumferential dissection, residual tumor due to complex tumor morphology, and esophagogastric reflux symptoms. She currently has no symptoms and no evidence of recurrence 1 year after surgery.

## 3 Discussion

The indication for surgical treatment for esophageal leiomyoma is when the patient is symptomatic, when the tumor is large or tends to grow, or when the tumor may have a malignant component. In cases of giant leiomyoma, esophagectomy is often performed in practice. Although the tumor shape was complex in this case, preoperative examinations revealed a smooth tumor surface and no adhesions with the surrounding tissues; thus, we opted for enucleation. However, we considered converting to esophagectomy if the tumor was directly damaged or there was significant destruction of the esophageal wall, especially the mucosal layer. The tumor was mainly located on the left side of the esophagus but the patient was operated on using a right thoracoscopic technique in the prone position, considering the possibility of conversion. One of the advantages of thoracoscopic surgery is the magnified operative view, which was also important for the recognition of the dissected layer around the tumor in this case. For tumor handling, the surrounding tissue attached to the tumor was grasped with forceps to avoid tumor damage by direct grabbing.

Although there are some reports of traction with a support thread directly over the tumor [7,8], we did not use this method to avoid tumor dissemination. Instead, we manipulated the tumor using an Endoloop. This facilitated the application of moderate tension and good surgical fields; in this case, although the tumor was large horseshoe-shaped, we were able to complete enucleation. We believe that a major factor of tumor enucleation was the preservation of the mucosa and muscular layer, especially the external longitudinal muscles. If the tumor does not invade the surrounding tissues, enucleation might be a viable surgical option regardless of its location in the thoracic esophagus. The difficulty in this technique was the limited mobility of the forceps inherent to thoracoscopic surgery. Recently, robot-assisted surgery has been spreading rapidly, and we strongly felt that it may be suitable for this surgical procedure.

## 4 Conclusion

Minimally invasive thoracoscopic enucleation may be a treatment option for even large esophageal leiomyomas.

The following is the supplementary data related to this article.

Supplementary Video S1 Supplementary Video S1

## Author contribution

Ishida, Kurahashi, and Shinohara contributed to the conceptualization of the operation and this paper. Ishida was the primary operative surgeon. Murakami and Ishida participated in data collection. All authors reviewed and approved the manuscript prior to journal submission. Ishida is the primary Guarantor of this paper.

## Consent

Written informed consent was obtained from the patient to undergo surgery and have the details of the case reported in writing, all in accordance with the Declaration of Helsinki. All information that might identify the patient's identity was omitted.

## Ethical approval

Since this is a case report, it was exempted from institutional review board approval.

## Funding

This research did not receive any specific grants from funding agencies in the public, commercial, or not-for-profit sectors.

## Conflict of interest statement

None.
